# The ABA-induced NAC transcription factor MdNAC1 interacts with a bZIP-type transcription factor to promote anthocyanin synthesis in red-fleshed apples

**DOI:** 10.1093/hr/uhad049

**Published:** 2023-03-15

**Authors:** Wenjun Liu, Zhuoxin Mei, Lei Yu, Tingting Gu, Zhiqiang Li, Qi Zou, Shuhui Zhang, Hongcheng Fang, Yicheng Wang, Zongying Zhang, Xuesen Chen, Nan Wang

**Affiliations:** National Key Laboratory of Crop Biology, College of Horticulture Science and Engineering, Shandong Agricultural University, Tai’an, Shandong 271018, China; National Key Laboratory of Crop Biology, College of Horticulture Science and Engineering, Shandong Agricultural University, Tai’an, Shandong 271018, China; National Key Laboratory of Crop Biology, College of Horticulture Science and Engineering, Shandong Agricultural University, Tai’an, Shandong 271018, China; College of Agricultural Science and Technology, Shandong Agricultural and Engineering University, Jinan, Shandong 250100, China; National Key Laboratory of Crop Biology, College of Horticulture Science and Engineering, Shandong Agricultural University, Tai’an, Shandong 271018, China; National Key Laboratory of Crop Biology, College of Horticulture Science and Engineering, Shandong Agricultural University, Tai’an, Shandong 271018, China; National Key Laboratory of Crop Biology, College of Horticulture Science and Engineering, Shandong Agricultural University, Tai’an, Shandong 271018, China; StateForestry and Grassland Administration Key Laboratory of Silviculture in the Downstream Areas of the Yellow River, College of Forestry, Shandong Agricultural University, Tai’an, Shandong 271018, China; State Key Laboratory of Crop Genetics and Germplasm Enhancement, Nanjing Agricultural University, Nanjing 210095, China; National Key Laboratory of Crop Biology, College of Horticulture Science and Engineering, Shandong Agricultural University, Tai’an, Shandong 271018, China; National Key Laboratory of Crop Biology, College of Horticulture Science and Engineering, Shandong Agricultural University, Tai’an, Shandong 271018, China; National Key Laboratory of Crop Biology, College of Horticulture Science and Engineering, Shandong Agricultural University, Tai’an, Shandong 271018, China

## Abstract

Anthocyanins are valuable compounds in red-fleshed apples. The MdMYB10 transcription factor is an important regulator of the anthocyanin synthesis pathway. However, other transcription factors are key components of the complex network controlling anthocyanin synthesis and should be more thoroughly characterized. In this study, we used a yeast-based screening technology to identify MdNAC1 as a transcription factor that positively regulates anthocyanin synthesis. The overexpression of *MdNAC1* in apple fruits and calli significantly promoted the accumulation of anthocyanins. In binding experiments, we demonstrated that MdNAC1 combines with the bZIP-type transcription factor *MdbZIP23* to activate the transcription of *MdMYB10* and *MdUFGT*. Our analyses also indicated that the expression of *MdNAC1* is strongly induced by ABA because of the presence of an ABRE *cis*-acting element in its promoter. Additionally, the accumulation of anthocyanins in apple calli co-transformed with *MdNAC1* and *MdbZIP23* increased in the presence of ABA. Therefore, we revealed a novel anthocyanin synthesis mechanism involving the ABA-induced transcription factor MdNAC1 in red-fleshed apples.

## Introduction

Plant flavonoids, which are produced by the phenylalanine metabolic pathway, have been divided into three main subclasses (i.e. Flavonols, Anthocyanins, and Procyanidins) [1]. Anthocyanins are important natural pigments responsible for the coloration of various plants, but they also attract insects and contribute to seed dispersal [2]. Moreover, they are crucial for plant resistance to biotic and abiotic stresses [3–5]. Anthocyanins are also free radical scavengers with anti-aging and anti-cancer properties and may be useful for preventing cardiovascular diseases and improving human immune functions [6–8]. The anthocyanins with beneficial effects on human health are mainly derived from fruits. Accordingly, anthocyanin synthesis has been investigated in various fruits, including apple [9], strawberry [10], grape [11], and blood orange [12].

Apple (*Malus domestica* Borkh.) fruits are popular worldwide because they are a rich source of easily absorbed nutrients. In recent years, red-fleshed apples have attracted increasing attention because of their bright red color and high anthocyanin contents. Previous studies demonstrated that proteins in three major families (MYB, bHLH, and WD40) regulate anthocyanin synthesis in diverse plant species by forming a conserved M-B-W ternary complex [13]. The MYB transcription factors are important regulators of the expression of anthocyanin synthesis-related genes [14]. For example, MdMYB1 participates in the synthesis of anthocyanins in the peel of white-fleshed apples [9]. In red-fleshed apples, *MdMYB10*, which is an allele of *MdMYB1*, encodes a regulator of anthocyanin biosynthesis [15]. Other transcription factors involved in anthocyanin biosynthesis in red-fleshed apples have also been identified and characterized [16, 17]. However, recent research indicated that flavonoid synthesis is not solely mediated by the M-B-W complex, with NAC, WRKY, and AP2/ERF protein families also involved in the production of flavonoids [18–20].

The NAC (NAM, ATAF1/2, and CUC2) proteins form one of the largest classes of transcription factors in plants. The N-terminal of NAC transcription factors is highly conserved and contains a NAC domain comprising approximately 150 amino acids, whereas the C-terminal has transcriptional regulatory regions that are highly diverse among species [21]. The conserved NAC domain is also a functional region that binds to DNA and other proteins. Some NAC proteins can form homodimers or interact with other NAC proteins to produce heterodimers [22, 23]. Several studies have shown that NAC transcription factors are involved in many processes related to plant growth and development, including stress responses, lateral root development, flower organ formation, senescence, and fruit development and ripening [24–29]. The roles of NAC transcription factors associated with anthocyanin synthesis have recently been determined for Arabidopsis [30, 31] and peach [18]. In apple, NAC transcription factors are reportedly involved in anthocyanin synthesis [32, 33]. These previous studies did not elucidate the effect of hormones on anthocyanin synthesis in red-fleshed apples.

Abscisic acid (ABA) is an important hormone for plants to resist environmental stress (e.g. drought, cold, and salinity) [34–36], while also affecting many other life processes [35, 37–40]. The interaction between SNF1-related protein kinase 2 (SNRK2) and protein phosphatase 2C (PP2C) is inhibited by ABA, thereby activating *SNRK2* [41]. The released *SNRK2* phosphorylates ABA-responsive element (ABRE)-binding factors (ABFs), which bind to *cis*-acting elements (ACGTGG/TC) to activate downstream gene expression [42, 43]. Through this ABA signaling pathway, the expression levels of multiple transcription factor genes are up- or down-regulated [44]. According to recent studies, ABA is closely related to the synthesis of anthocyanins in diverse species [45–49]. However, the molecular mechanism underlying the effects of NAC family transcription factors on ABA-induced anthocyanin synthesis has not been thoroughly characterized in red-fleshed apples.

In this study, we screened a yeast one-hybrid library and then cloned and identified *MdNAC1*, which encodes a positive regulator of anthocyanin synthesis in red-fleshed apples. Specifically, MdNAC1 enhances anthocyanin production by interacting with the basic leucine zipper (bZIP) family member MdbZIP23 to form the MdNAC1–MdbZIP23 complex that significantly increases the transcription of downstream target genes, especially in the presence of ABA, implying MdNAC1 is highly responsive to ABA. These molecular changes ultimately lead to the accumulation of anthocyanins in apple fruits. The findings of this study reflect the potential physical interaction between NAC and bZIP transcription factors in a new molecular mechanism promoting anthocyanin synthesis in the ABA signaling pathway.

## Results

### MdNAC1 binds to the *MdMYB10* promoter

**Figure 1:**
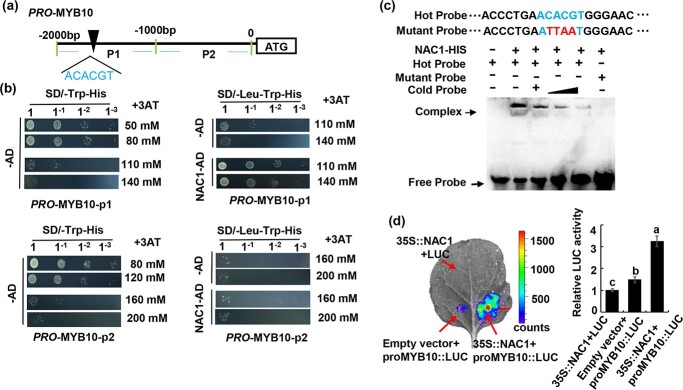
MdNAC1 binds MdMYB10 promoter and promotes its transcriptional expression. (a) Location analysis of NAC binding cis-acting elements in *MdMYB10* promoter sequence. The *MdMYB10* promoter sequence was divided into P1and P2 fragments and inserted into pHIS2 vector. (b) Yeast one hybrid test showed that MdNAC1 could interact with P1 fragment of *MdMYB10* promoter sequence. (c) Electrophoretic mobility shift assays revealed that MdNAC1 binds to the [-CACG-] motif in the *MdMYB10* promoter. Hot probe is an abiotin labeled fragment containing [CACG] motif. The cold probe is an unlabeled competitive probe (100 times the concentration of the hot probe). The mutation probe contains four nucleotide substitutions. (d) The effect of MdNAC1 on the activity of *MdMYB10* promoter was confirmed by the doubleluciferase report experiment in tobacco leaves. Values are means ±SD of three independent biological replicates, different letters indicate significant differences (p < 0.05). Scale bar represents 1 cm.

Earlier research confirmed that MdMYB10 is a major regulator of anthocyanin synthesis in red-fleshed apples [15]. Hence, the transcription factors functioning upstream of *MdMYB10* should be explored. Transcription factors from different families can specifically bind to various *cis*-acting elements in the *MdMYB10* promoter to regulate anthocyanin synthesis [50, 51]. Using the PlantCARE website (https://bioinformatics.psb.ugent.be/webtools/plantcare/html),we analysed the *MdMYB10* promoter sequence and identified several important *cis*-acting elements (Fig. S1, see online supplementary material). The *MdMYB10* promoter was then used as the bait to screen a yeast one-hybrid cDNA library, which revealed MdNAC1 (280 amino acid protein) can interact with the *MdMYB10* promoter (Fig. S2, Table S1, see online supplementary material). We also detected an interaction with the *MdWRKY11* transcription factor, which was in accordance with the results of our previous study [50]. Because no other transcription factors able to interact with the *MdMYB10* promoter were detected, we focused on MdNAC1. To validate the binding of MdNAC1 to the *MdMYB10* promoter, we divided the promoter into two fragments (P1 and P2), which were inserted into the pHIS2 vector for a yeast one-hybrid analysis (Fig. 1a and b). The results indicated that MdNAC1 can interact with P1, but not P2. An earlier investigation demonstrated that NAC family proteins can specifically bind to -CACG-containing *cis*-acting elements [23]. By analysing the *cis*-acting elements in the *MdMYB10* promoter, we detected a NAC-specific binding motif (ACACGT) (Fig. 1a). The electrophoretic mobility shift assay (EMSA) results confirmed that MdNAC1 can bind to this site (Fig. 1c). Increases in the concentration of the unlabeled competitor probe gradually decreased the observed binding. Replacing the ACACGT sequence with ATTAAT eliminated the binding, even in the absence of the competitor probe. To explore the effect of the binding on *MdMYB10* transcription, we performed a transient LUC complementation imaging analysis using tobacco leaves (Fig. 1d and e). The luminescence intensity resulting from the co-expression of MdNAC1–62-SK and proMdMYB10–0800-LUC was significantly higher than that of the negative control. These results indicate that MdNAC1 can activate the transcription of *MdMYB10* by binding directly to its promoter.

### Bioinformatics analysis and determination of the autoactivation of MdNAC1

**Figure 2:**
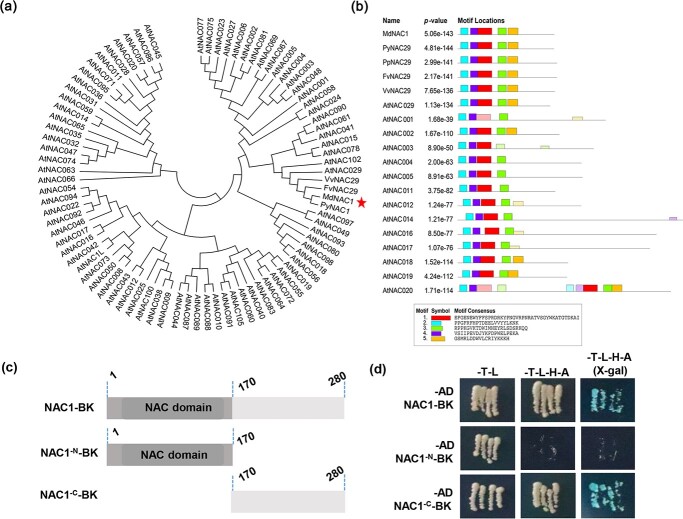
MdNAC1 Bioinformatics Analysis and Self Stimulating Activity Identification. (a) The evolutionary tree analysis of apple MdNAC1 and other species shows that the branch of MdNAC1 is marked by a red pentagram. (b) Protein domain analysis of MdNAC1 and other species. Different colored rectangles represent different but conservative structural domains. (c) The schematic diagram of MdNAC1 protein segment. The full-length CDS of NAC1 was divided into NAC1^-N^ and NAC1^-c^ fragments. Different numbers represent the number of amino acids. (d) Self activation activity analysis of MdNAC1 protein. NAC1^-c^ was verified to have self activating activity in the T-L-H-A (X-gal) screening medium.

We constructed a phylogenetic tree for NAC1 in apple and other species (Fig. 2a), which revealed the high homology between apple and pear NAC1 sequences, which were very similar to AtNAC029 in Arabidopsis. A bioinformatics analysis indicated the NAC1 N-terminal is more highly conserved than the C-terminal in various species (Fig. S3, see online supplementary material). The conserved NAC domain in the N-terminal has binding activity and was divided into A, B, C, D, and E subdomains [21]. Consistent with the results of previous studies, we determined that most NAC family proteins contain five conserved domains. However, unlike domains A, B, C, and D, the E domain is lacking in some proteins (Fig. 2b). To examine the self-activation of MdNAC1, we divided MdNAC1 into two fragments (NAC1^-N^-BKT7 and NAC1^-C^-BKT7) for a yeast two-hybrid (Y2H) analysis involving the full-length MdNAC1 sequence (Fig. 2b and c). Y2H results showed that the self-activation of the complete MdNAC1 sequence was the same as that of the C-terminal fragment, but this self-activation was undetectable when the C-terminal was deleted. These findings suggest MdNAC1 may activate the expression of downstream target genes.

### MdNAC1 promotes anthocyanin accumulation

**Figure 3:**
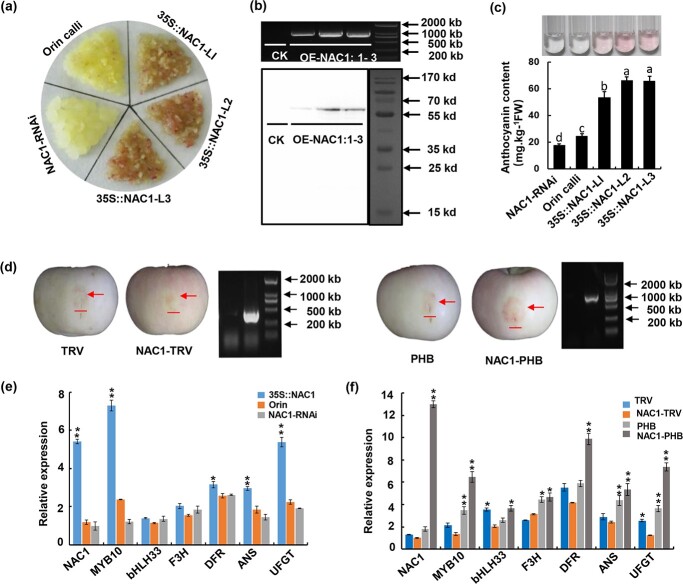
Overexpression of MdNAC1 promoted the accumulation of anthocyanins in apple callus and fruit. (a) The callus of wild type ‘Orin’, *MdNAC1* overexpression transgenic line (three independent lines L1 - L3) and *MdNAC1* silencing transgenic line NAC1-RNAi were under light conditions. After 10 days of normal growth under dark conditions, the cells were transferred to light conditions for 5-7 days. (b) The existence of stable transgenes in L1 - L3 callus was confirmed by PCR amplification and western blotting of GFP antibody. (c) The anthocyanin content in Orin, 35S:: NAC1 (L1-L3) and NAC1-RNAi callus was determined by spectrophotometry. According to one-way analysis of variance (ANOVA), different letters indicate significant differences (p < 0.05). (d) ‘Fuji’ apple was used for transient injection experiment, NAC1-TRV2 and TRV1 were injected into apple skin in equal proportion to inhibit *NAC1* expression, and the existence of NAC1 transgene was verified by PCR amplification. NAC1-PHB and P19 were mixed and injected into apple skin in equal proportion for transient overexpression. PCR amplification verified the existence of transgene. Scale bar represents 1 cm. (e) Transcription level of *NAC1* and anthocyanin synthesis pathway related genes in Orin, 35S:: NAC1 and NAC1-RNAi callus. Values are means ±SD of three independent biological replicates. Asterisks denote t - test significance: ^*^P < 0.05 and ^**^P < 0.01. (f) Transcription level of *NAC1* and anthocyanin synthesis related genes in TRV NAC1-TRV PHB NAC1-PHB apple peel tissue. Values are means ±SD of three independent biological replicates. Asterisks indicate statistical significance by Tu key’s test using DPS software (^**^P < 0.01).

Because MdNAC1 was observed to transcriptionally activate *MdMYB10* (Fig. 1), we speculated that MdNAC1 may be involved in anthocyanin synthesis. To assess whether MdNAC1 contributes to anthocyanin synthesis, we transformed ‘Orin’ apple calli with *MdNAC1* (Fig. 3a)*.* Three independent *MdNAC1*-overexpressing ‘Orin’ apple callus lines (L1, L2, and L3) were obtained. Additionally, we inserted the sequence encoding the MdNAC1 C-terminal into the *pFGC-1008* vector to construct the recombinant NAC1-RNAi plasmid, which was incorporated into apple calli to obtain the NAC1-RNAi strain (Fig. 3a). The presence of the transgene was verified by RT-qPCR and western blot analyses (Fig. 3b). In contrast to the wild-type and NAC1-RNAi calli, the *MdNAC1*-overexpressing calli were pink because of the accumulation of anthocyanins following the incubation under light. The spectrophotometric analysis of the extracts showed that the anthocyanin content in the *MdNAC1*-overexpressing calli was 2.2 to 3.3 times higher than that in the wild-type calli and 3.0 to 4.5 times higher than that in the NAC1-RNAi calli (Fig. 3c). Next, the expression levels of genes involved in anthocyanin biosynthesis in the *MdNAC1*-overexpressing, NAC1-RNAi, and wild-type calli were analysed by RT-qPCR (Fig. 3e). Compared with the NAC1-RNAi and wild-type calli, the expression levels of genes encoding anthocyanin synthesis-related transcription factors and enzymes (*MdMYB10*, *MdbHLH3*, *MdF3H*, *MdDFR*, *MdANS*, and *MdUFGT*) were higher in the *MdNAC1*-overexpressing calli; the differences were significant for *MdMYB10* and *MdUFGT*. To further verify that MdNAC1 affects anthocyanin synthesis in apple fruits, we constructed MdNAC1-PHB (gene overexpression) and MdNAC1-TRV (virus-induced gene silencing) recombinant plasmids for the *Agrobacterium tumefaciens*-mediated transient transformation experiments (Fig. 3d). The overexpression of *MdNAC1* in apple fruits significantly increased the anthocyanin content in the peel, whereas the opposite results were found after MdNAC1 was silenced. On the basis of the RT-qPCR analysis, all of the anthocyanin synthesis-related gene expression levels increased in response to the overexpression of *MdNAC1* (Fig. 3f). The silencing of *MdNAC1* tended to decrease the expression of these anthocyanin synthesis-related genes.

### MdNAC1 binds directly to the anthocyanin synthesis pathway structural gene *MdUFGT*

**Figure 4:**
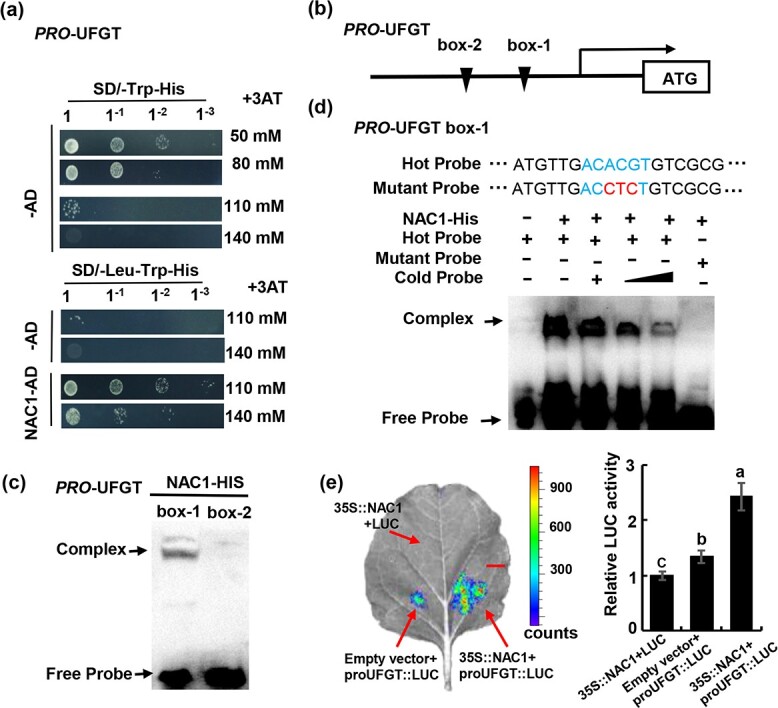
MdNAC1 directly binds to the MdUFGT promoter to promote anthocyanin synthesis. (a) The yeast one hybrid test showed that *MdNAC1* could interact with the MdUFGT promoter. (b) The MdUFGT promoter sequence contains two NAC specific binding elements, which are located at -870bp and -904bp positions upstream of ATG. (c) The EMSA experiment proves that MdNAC1 can bind to box-1 but not box-2. (d) MdNAC binds the [CACG] motif in the *UFGT* promoter sequence through the EMSA experiment. Mutation and competition probes were added according to different combinations. The competitive probe is 100 times more than the Hort probe, and the mutant probe has replaced three nucleotide sequences. (e) Transcriptional activation of MdNAC1 on *MdUFGT* promoter was confirmed by double luciferase reporter in tobacco leaves. different letters indicate significant differences (p < 0.05). Scale bar represents 1 cm.

In the apple calli and fruits overexpressing *MdNAC1*, the expression levels of the anthocyanin biosynthesis-related genes were generally up-regulated, especially *MdMYB10* and *MdUFGT* (Fig. 3d and f; Fig. S4, see online supplementary material). The increase in *MdMYB10* expression was due to the binding of MdNAC1 to the *MdMYB10* promoter. However, whether MdNAC1 can also bind to the *MdUFGT* promoter remained to be verified. Therefore, we performed a Y1H assay (Fig. 4a). Unlike the control, co-transformed with MdNAC1-pGADT7 and MdUFGT-pHIS2 grew normally on the screening medium lacking -Leu, −Trp, and -His, indicative of the interaction between MdNAC1 and the *MdUFGT* promoter. By cloning and analysing the *MdUFGT* promoter sequence, we identified two specific NAC-binding sites (Fig. 4b). Notably, the overexpression of *MdNAC1* also resulted in increased *MdANS*, *MdDFR*, and *MdF3H* expression levels. Thus, *cis*-acting elements in promoters of MdANS, MdDFR and MdF3H were analysed. Unfortunately, NAC-binding elements were not identified in these three promoters. The EMSA results proved that MdNAC1 can bind directly to box1 of the *MdUFGT* promoter, but not to box 2 (Fig. 4c). We designed competitor and mutant probes for box 1. Along with the hot probe, different concentrations of the competitor probe were added. The observed binding decreased as the competitor probe concentration increased. When the hot probe was replaced by the mutant probe, the binding was undetectable (Fig. 4d). The luciferase reporter experiments demonstrated that MdNAC1 can significantly activate the *MdUFGT* promoter (Fig. 4e and f). Considered together, these findings imply that MdNAC1 enhances anthocyanin synthesis by binding directly to the *MdUFGT* promoter, thereby increasing gene expression.

### MdNAC1 interacts with MdbZIP23

**Figure 5:**
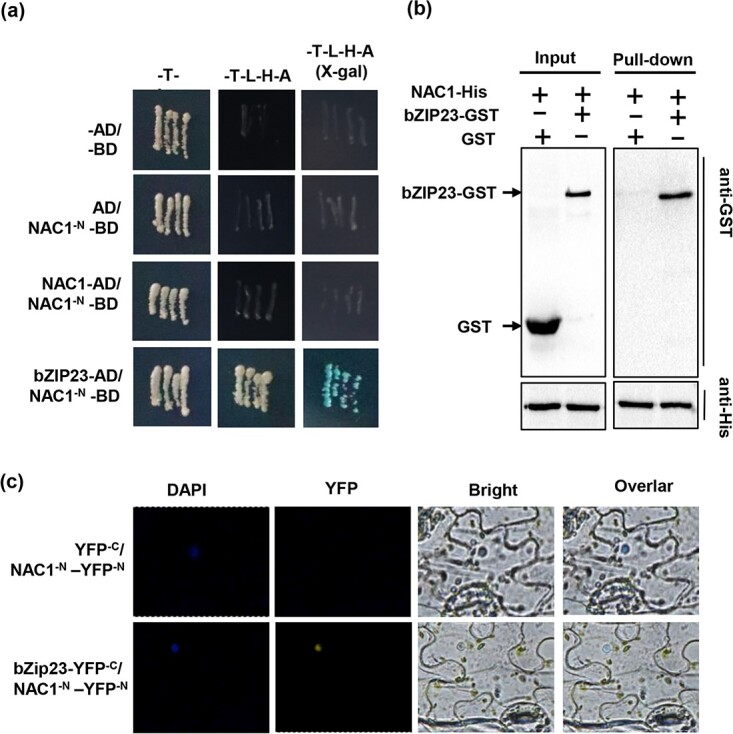
MdNAC1 and MdbZIP23 can interact with each other. (a) Yeast two hybrid showed that MdNAC1 can not form homodimer, but can interact with MdbZIP23. Yeast two hybrid showed that NAC1^-N^ (1-170) sequence had no self activating ability and could not form homodimer with the full length of MdNAC1. When MdbZIP23 was added, it could grow normally in the -T-L-H-A deletion medium. (b) Pull down assay proved that MdNAC1 can interact with MdbZIP23 in vitro. NAC1-HIS and bZIP23-GST protein was mixed and purified. Western blotting was performed with GST antibody. bZIP23-GST can be pulled down by NAC1-HIS, but empty GST can not. (c) Bifc analysis showed the interaction between MdNAC1 and MdbZIP23 in vivo. Bifc analysis showed the interaction between MdNAC1 and MdbZIP23 in vivo. DAPI can determine the nuclear position of the nucleus. The yellow fluorescent signal indicates that two genes interact, and overlar refers to the merging channel.

A previous study showed that NAC proteins form homodimers through their conserved NAC domains [23]. To assess whether MdNAC1 can also form homodimers, we constructed MdNAC1-pGADT7 and MdNAC1^-N^-pGBKT7 recombinant plasmids for yeast two-hybrid analyses (Fig. 5a). The yeast two-hybrid results suggested that MdNAC1 cannot form homodimers. Next, we attempted to identify other transcription factors that can interact with MdNAC1. Specifically, we used the MdNAC1^-N^ sequence as the bait to screen for potential interacting proteins (Table S2, see online supplementary material). Several candidate proteins were analysed in yeast two-hybrid experiments, which confirmed that the bZIP family protein MdbZIP23 (431 amino acids) can interact with MdNAC1^-N^ (Fig. 5a;Fig. S5, see online supplementary material). Other transcription factors, including *MdMYB14* and *MdERF113*, were not considered further because they did not interact with MdNAC1. Similarly, the anthocyanin synthesis-related transcription factors *MdMYB10* and *MdbHLH33* were also unable to interact with MdNAC1. To further verify the interaction between MdNAC1 and MdbZIP23, we purified MdNAC1-His and MdbZIP23-GST, which were expressed in a prokaryotic system, for pull-down assays (Fig. 5b). The yeast two-hybrid and pull-down assays demonstrated that MdNAC1 can interact with MdbZIP23 in vitro, while the bimolecular fluorescence complementation experiments indicated that MdNAC1 can interact with MdbZIP23 *in vivo* (Fig. 5c).

### The MdNAC1–MdbZIP23 complex promotes anthocyanin synthesis

**Figure 6:**
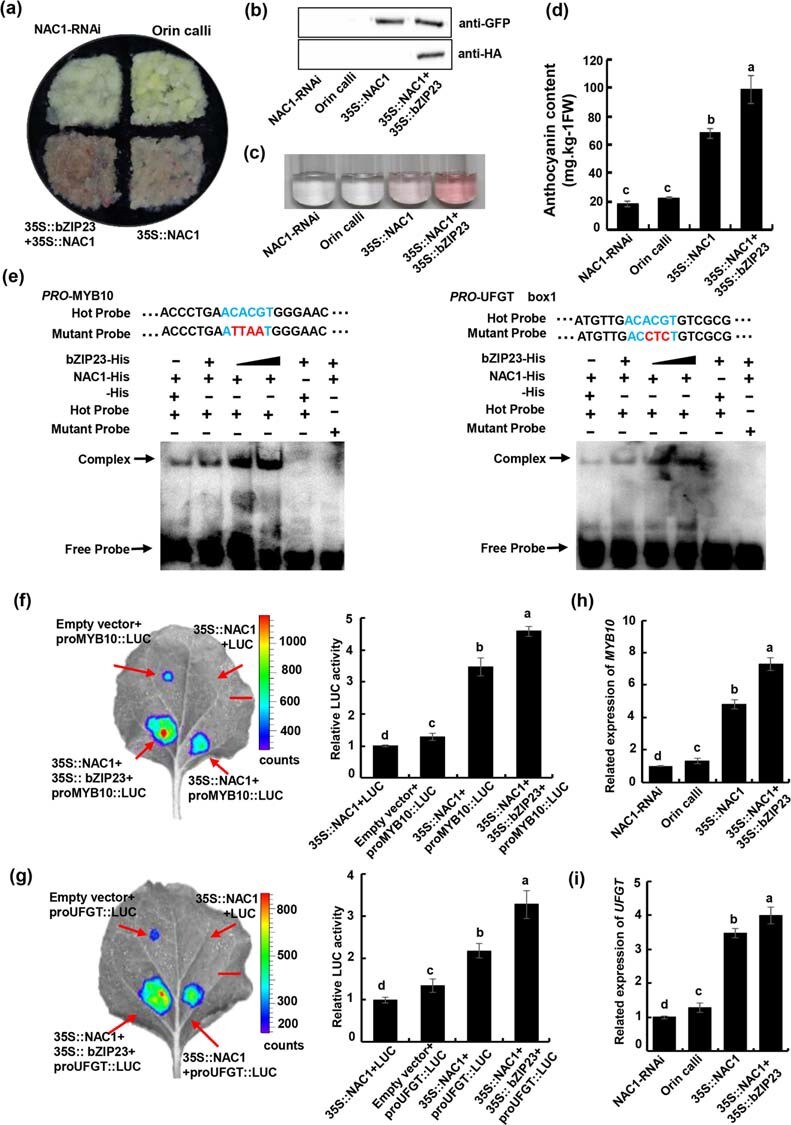
MdNAC1 interacts with MdbZIP23 to promote anthocyanin synthesis. (a) The state of wild type “Orin”, MdNAC1-RNAi, 35S:: MdNAC1 and 35S:: MdNAC1+bZIP23 callus under light conditions. (b) GFP antibody was used in western blotting to prove the existence of stable transgene. (c) The anthocyanin content in the callus was extracted with 1% hydrochloric acid methanol solution, and it was extracted for 24 hours under 4 degrees of darkness. (d) The content of anthocyanin in callus was determined by spectrophotometry, Values are means ±SD of three independent biological replicates, different letters indicate significant differences (p < 0.05). (e) EMSA slightly modified the method to prove that the addition of bZIP23 promoted the binding of MdNAC1 to *MdMYB10* and *MdUFGT*. On the basis of single addition of NAC1-His protein, bZIP23-His protein was added and gradually increased, and empty His protein was added to the control group. (f)-(g) The luciferase report experiment in tobacco showed that the addition of bZIP23 enhanced the transcriptional activation of *NAC1* on downstream genes, different letters indicate significant differences (p< 0.05). Scale bar represents 1 cm. (h)-(i) The expression level of *MdMYB10* and *MdUFGT* in different calli was determined by qRT-PCR. Values are means ±SD of three independent biological replicates, different letters indicate significant differences (p < 0.05).

Because MdNAC1 and MdbZIP23 can combine to form a complex, we investigated whether this complex influences anthocyanin synthesis. We generated transgenic calli carrying 35::NAC1 + 35::bZIP23 (Fig. 6a). The presence of the transgenes was confirmed using different labeled antibodies (Fig. 6b). In response to a light treatment, the calli with 35::NAC1 + 35::bZIP23 accumulated more anthocyanins than the calli with 35::NAC1 alone, implying that the MdNAC1–MdbZIP23 complex further promoted the accumulation of anthocyanins (Fig. 6c and d). The effects of the MdNAC1–MdbZIP23 complex on the *MdMYB10* and *MdUFGT* promoters were clarified on the basis of an EMSA involving MdNAC1-His and MdbZIP23-His (Fig. 6e). The His-tag was used as a control to ensure the same amount of protein was added to each group. When MdbZIP23-His was added, the binding strength of MdAC1 with MdMYB10 and MdUFGT promoter increased significantly, suggesting that MdbZIP23 formed a complex with MdNAC1 to enhance the subsequent binding to downstream genes. To analyse the ability of the complex to activate target gene transcription, two recombinant plasmids (MdNAC1–62-SK and MdbZIP23–62-SK) were used together in transient LUC complementation imaging experiments (Fig. 6f and g). The luminescence intensity was greater for 35S::NAC1 + 35S::bZIP23 with proMYB10::LUC than for 35S::MdNAC1 with proMYB10::LUC. The same results were obtained when the MdUFGT promoter was integrated into the pGreenII 0800-LUC vector as a reporter. Finally, the transcription levels of the anthocyanin biosynthesis-related genes in calli were analysed by RT-qPCR (Fig. 6h and k; Fig. S6, see online supplementary material). Compared with the NAC1-RNAi and wild-type calli, the *MdMYB10*, *MdbHLH33*, *MdF3H*, *MdDFR*, and *MdUFGT* expression levels were higher in the 35S::NAC1 calli. These genes were expressed at even higher levels in the calli carrying 35S::NAC1 + 35S::bZIP23. These results indicate that MdNAC1 interacts with MdbZIP23 and promotes the transcription of downstream genes to increase the accumulation of anthocyanins.

### Abscisic acid induces *MdNAC1* expression

**Figure 7:**
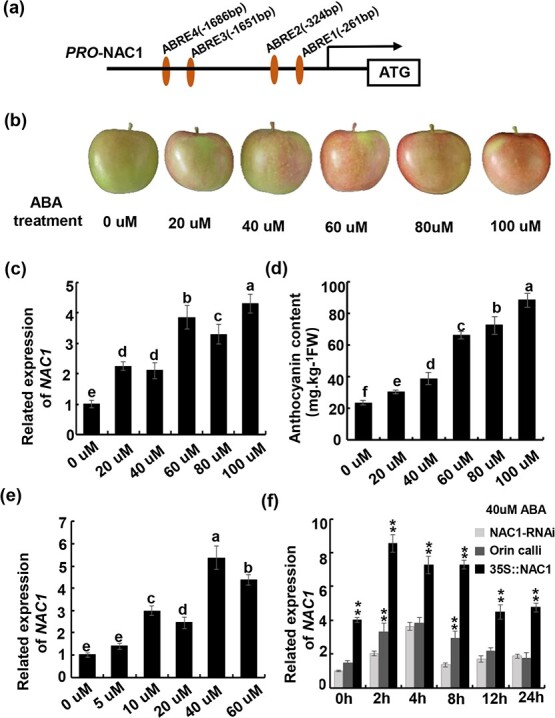
MdNAC1 expression was induced by ABA in apple fruit and callus. (a) *MdNAC1* promoter sequence contains four ABA binding elements (named ABRE1-4 ), which are located at positions -261bp, -324bp, -1651bp and -1686bp upstream of ATG. (b) ‘Fuji’ apples were immersed in ABA solution of different concentrations for one minute, dried naturally and then transferred to light for 3-5 days. (c) The expression level of *MdNAC1* in apple meat treated with different concentrations of ABA was determined by RT-qPCR. Values are means ±SD of three independent biological replicates. Different letters indicate significant differences (p < 0.05). (d) Anthocyanin accumulation in apple peel treated with ABA at different concentrations. Different letters indicate significant differences (p < 0.05). (e) RT-qPCR was used to determine the transcription level of MdNAC1 in Orin apple calli treated with ABA at different concentrations. Statistical significance:(P < 0.05). (f) The expression of *MdNAC1* in NAC1-RNAi, Orin, 35S:: NAC1 callus treated with 40μM ABA was determined by RT-qPCR. Each value has three biological repeats.Asterisks indicate statistical significance by Tukey’ s test using DPS software (^**^P < 0.01 ).

The effect of ABA on anthocyanin accumulation has been reported in different species [45–49, 52]. In the current study, four ABRE *cis*-acting elements were detected by cloning and analysing the *MdNAC1* promoter (Fig. 7a). To explore whether *MdNAC1* expression is induced by ABA, ‘Fuji’ apple fruits were treated with different concentrations of ABA (Fig. 7b). Compared with the untreated fruits, the *MdNAC1* expression level increased significantly as the ABA concentration increased, reflecting the inductive effects on *MdNAC1* expression in apple fruits (Fig. 7c). Notably, increases in the ABA concentration were associated with increases in the anthocyanin contents in apple peels (Fig. 7b and d). The anthocyanin content also gradually increased in the ‘Orin’ calli in response to increasing ABA concentrations, with peak levels detected after the 40 μM ABA treatment (Figs S7 and S8, see online supplementary material). To determine whether *MdNAC1* expression was induced by ABA in ‘Orin’ calli, we completed an RT-qPCR analysis, which revealed that *MdNAC1* was more highly expressed in the calli treated with different ABA concentrations than in the untreated calli (Fig. 7e). The *MdNAC1* expression level was highest following the 40 μM ABA treatment. We then treated the ‘Orin’，NAC1-RNAi calli and 35S::NAC1 calli with 40 μM ABA and measured the *MdNAC1* expression level 24 h later (Fig. 7f). The data indicated that the expression of MdNAC1 in all calli increased first and then decreased. However, ABA-induced *MdNAC1* expression appeared to be higher in the 35S::NAC1 calli than in the ‘Orin’ calli and NAC1-RNAi calli. These results indicate that *MdNAC1* expression in apple fruits is strongly induced by ABA.

### Abscisic acid induces the MdNAC1–MdbZIP23 complex to promote anthocyanin accumulation

**Figure 8:**
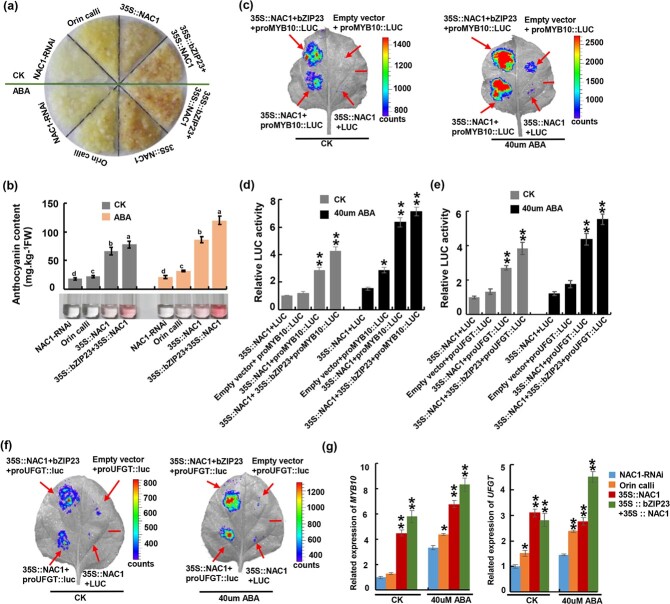
MdNAC1 interacts with MdbZIP23 to further promote anthocyanin accumulation in the presence of ABA. (a) The condition of different calli under light conditions. No ABA was added above the split line, and 40μM ABA was added below the split line. They were all transferred to light for 5-7 days after 10 days of growth in dark conditions.(b) Anthocyanin content in callus determined by spectrophotometry, gray means no ABA is added in the callus, orange means 40μM ABA is added. Values are means ±SD of three independent biological replicates. Different lowercase letters indicate significant differences (P < 0.05). (c-f) The deformation of the transient luciferase report experiment in tobacco leaves proved that MdNAC1 increased the transcriptional activation of *MdMYB10* and *MdUFGT* in the presence of ABA. Tobacco leaves were sprayed with ABA after injection, and leaves without ABA were used as control. Values are means ±SD of three independent biological replicates. Asterisks indicate statistical significance by Tu key’s test using DPS software (^**^P < 0.01 ). Scale bar represents1 cm. (g) MdNAC1 promotes the transcription level of *MdMYB10* and *MdUFGT*, and the co-expression of *MdNAC1* and *MdbZIP23* further enhances the transcription level of *MdMYB10* and *MdUFGT* when ABA appears. Values are means ±SD of three independent biological replicates. Asterisks indicate statistical significance by Tu key's test using DPS software (^*^P < 0.05 and ^**^P < 0.01.).

To elucidate the effects of the MdNAC1–MdbZIP23 complex on anthocyanin levels in the presence of ABA, we examined the effects of exogenously applied ABA on anthocyanin synthesis in transgenic apple calli (Fig. 8a and b). In the absence of exogenous ABA, the anthocyanin content in the 35S::NAC1 + 35S::bZIP23 calli was 1.5 times higher than that in the 35S::NAC1 calli, 2.3 times higher than that in the ‘Orin’ calli, and 2.9 times higher than that in the NAC1-RNAi calli. After the exogenous ABA treatment, the anthocyanin contents increased in the ‘Orin’, 35S::NAC1, and 35S::NAC1 + 35S::bZIP23 calli, with the largest increase detected in the 35S::NAC1 + 35S::bZIP23 calli. The increase in the anthocyanin content in the NAC1-RNAi calli was not significant. To clarify whether the ABA treatment affected the binding of MdNAC1 to the downstream target genes, we performed luciferase reporter experiments involving ABA (Fig. 8c–f). The results showed that when 35S::NAC1 and proMYB10::LUC were injected into tobacco, the application of ABA resulted in a significant increase in the luminescence intensity. Additionally, the luminescence intensity was greater in tobacco infiltrated with 35S::NAC1 + 35S::bZIP23 and proMYB10::LUC than in tobacco carrying 35S::NAC1 alone. Similar results were observed for the ABA-treated tobacco infiltrated with 35S::NAC1 + proUFGT::LUC and 35S::NAC1 + 35S::bZIP23 + proUFGT::LUC. These findings suggest that ABA can promote *MdNAC1* expression and enhance the transcription of the downstream target genes *MdMYB10* and *MdUFGT*. Moreover, the addition of MdbZIP23 further promotes the transcriptional activation of *MdMYB10*. This was supported by our RT-qPCR analysis, which detected increased *MdMYB10* and *MdUFGT* expression levels in the presence of ABA (Fig. 8g). Furthermore, the transcription levels of the genes related to anthocyanin synthesis were also up-regulated in response to the ABA treatment (Fig. S9, see online supplementary material). Hence, ABA induces the interaction between MdNAC1 and MdbZIP23 and the resulting complex promotes the transcription of the downstream target genes, ultimately leading to the accumulation of anthocyanins in apple fruits.

## Discussion

The MYB–bHLH–WD40 ternary complex plays an important role in the synthesis of anthocyanins or procyanidins in different species [53, 54]. The MYB transcription factor is the main component determining the specificity of the complex. Several studies verified the key regulatory functions of MYB transcription factors during anthocyanin synthesis in diverse species, including PAP1 in Arabidopsis [55], AN2 in petunia [56], and MYB10 and MYB114 in pear [57, 58]. Among the MYB family members, the expression of *MdMYB10* directly affects anthocyanin accumulation in red-fleshed apples [15, 59]. Different transcription factors can regulate the expression of *MdMYB10* by binding to specific *cis*-acting elements in its promoter. In the present study, we identified a NAC family transcription factor in apple (MdNAC1) that can promote anthocyanin synthesis by inducing *MdMYB10* expression. This pattern of anthocyanin accumulation by promoting MYB10 expression is similar to previous findings [19, 51]. In addition, by screening a yeast cDNA library, we confirmed the results of earlier research that identified MdWRKY11 as an upstream regulator of *MdMYB10*. Unfortunately, we did not detect HY5, which is widely recognized as a photoresponsive factor regulating anthocyanin synthesis [60, 61].

The NAC proteins are a relatively new class of transcription factors mediating anthocyanin synthesis. In blood-fleshed peach, PpNAC1 can form a heterodimer with BL to promote the transcription of *PpMYB10.1* and the accumulation of anthocyanins [18]. In Arabidopsis, the overexpression of ANAC078 can promote the accumulation of flavonoids [30]. In apple, MdNAC52 binds to MdMYB9/MdMYB11, which then modulates gene expression to increase anthocyanin and procyanidin contents [32]. Moreover, the overexpression of *MdNAC42* in apple calli can significantly induce anthocyanin accumulation [33]. In this study, we determined that MdNAC1 is a transcription factor that can bind directly to the *MdUFGT* and *MdMYB10* promoters, with the resulting changes in gene expression leading to increased anthocyanin contents. Conversely, anthocyanin accumulation decreased significantly following the inhibition of MdNAC1 in apple calli and fruits (Fig. 3). In an earlier study, apple seedlings overexpressing *MdNAC1* exhibited dwarfism [62]. In addition, the overexpression of *MdNAC1* can also increase the drought tolerance of plants [63]. Generally, plants accumulate flavonoids in response to drought stress. However, whether MdNAC1 can promote anthocyanin synthesis remained unknown. The strong self-activation of NAC transcription factors detected in this study was in accordance with the findings of previous investigations [62]. This self-activation may increase the transcriptional activation of the downstream genes, which may help to explain the observed increase in the expression of the downstream genes following the overexpression of *MdNAC1*.

The synergistic effects of multiple transcription factors are important for enhancing anthocyanin synthesis in plants. In this experiment, we screened a yeast cDNA library and detected a bZIP family transcription factor (*MdbZIP23*) that interacts with MdNAC1. In Arabidopsis, a NAC transcription factor (*ANAC096*) and the bZIP family protein ABF2 synergistically activate *RD29A* transcription [64]. Similarly, compared with the effects of MdNAC1 alone, the interaction between MdNAC1 and MdbZIP23 resulted in higher *MdMYB10* and *MdUFGT* expression levels, which ultimately led to further increases in the anthocyanin contents of apple calli. The bZIP transcription factors are important for plant growth and development, resistance to abiotic stresses, and responses to hormone signals [65–69]. Recent studies demonstrated that the bZIP family is also involved in anthocyanin synthesis. For example, HY5 can bind directly to structural genes to promote anthocyanin synthesis in tomato [70], whereas it activates the MYB75/PAP1 transcription factor in Arabidopsis [71]. In apple, bZIP44 can interact with MYB1 to promote anthocyanin production [72]. Our results imply that MdbZIP23 can indirectly promote the synthesis of anthocyanins by forming a complex with MdNAC1. Whether MdbZIP23 can directly promote anthocyanin synthesis remains to be investigated.

Plant hormones, such as ABA, ethylene, jasmonate, and auxin, influence anthocyanin synthesis [73–76]. Generally, ABA was considered as a plant defense hormone mainly involved in response to abiotic stress [35], but recent studies have generated increasing evidence that ABA can also modulate anthocyanin contents [49，53]. In this study, we detected several ABA-related *cis*-acting elements in the *MdNAC1* promoter. After treating apple fruits and calli with different concentrations of ABA, the *MdNAC1* transcription level increased, with peak levels detected earlier in 35S::NAC1 samples than in the wild-type controls, possibly because the overexpression of *MdNAC1* increased the sensitivity to ABA. Furthermore, in response to the ABA treatment, the *MdNAC1* expression level was lower in the NAC1-RNAi calli than in the ‘Orin’ calli, suggesting the inhibition of MdNAC1 decreases the ability of plants to perceive ABA. These experimental findings indicate that *MdNAC1* expression is induced by ABA and is important for the ABA signaling pathway in plants (Fig. 7). Moreover, we observed that the interaction between MdbZIP23 and MdNAC1 enhances the transcriptional activation of *MdMYB10* and *MdUFGT* and increases anthocyanin synthesis in the presence of ABA. Notably, the expression of *MdMYB10* in the ‘Orin’ and NAC1-RNAi calli increased following the ABA treatment, which may be related to the fact that the *MdMYB10* promoter contains ABRE *cis*-acting elements (Fig. 8g; Fig. S1, see online supplementary material). Although the *MdMYB10* transcription level was relatively high in the NAC1-RNAi and ‘Orin’ calli, it did not reach the level detected in the 35S::NAC1 calli.

**Figure 9:**
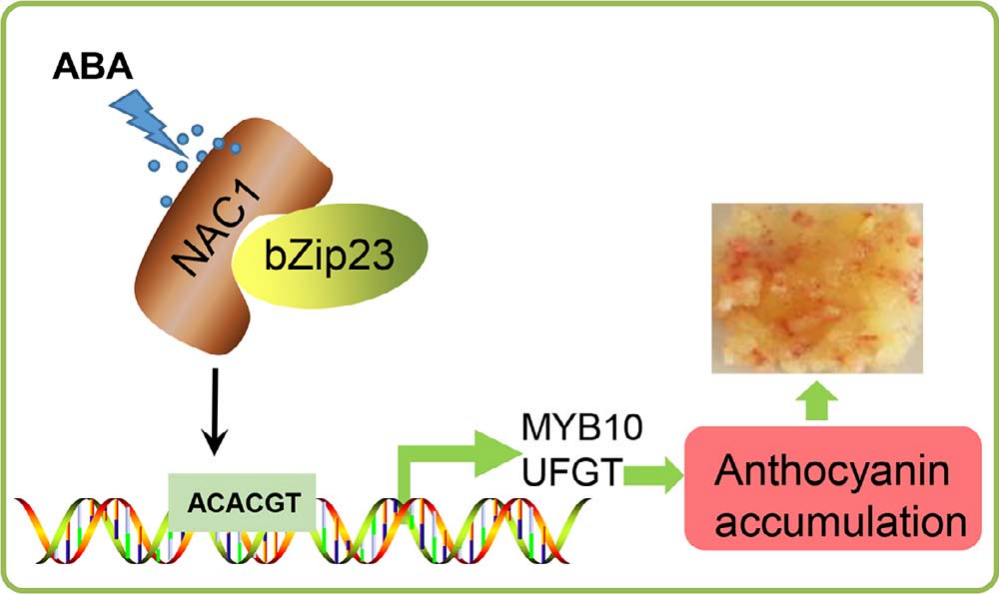
A simplified model of ABA induction of anthocyanin synthesis by MdNAC1.

Fig. 9 presents a schematic diagram summarizing the effects of MdNAC1 on anthocyanin synthesis. Specifically, MdNAC1 promotes the expression of *MdMYB10* and *MdUFGT* and the accumulation of anthocyanins by forming a complex with MdbZIP23 in the ABA signaling pathway. In conclusion, our study revealed a novel MdNAC1 function related to anthocyanin synthesis, thereby enriching our understanding of the network regulating anthocyanin biosynthesis. The data generated in this study may be relevant for exploiting the effects of ABA on anthocyanin synthesis to optimize the coloration of red-fleshed apple fruits.

## Materials and methods

### Plant materials

‘Orin’ apple calli, which were used for transformations, were grown aseptically on Murashige and Skoog (MS) solid medium. ‘Orin’ calli were propagated for one generation every 15 days. The hormone content of the medium and the culture conditions were as previously described [77]. The apple injection experiment was performed using ‘Fuji’ apples from trees grown at the experimental station of the National Apple Engineering Technology Research Center, Tai’an city, Shandong province, China. ‘Fuji’ apples that are about to mature but have not removed the fruit bag were collected on 21 September.

### Yeast one-hybrid library screening and yeast one-hybrid assay

We collected the flesh of ‘Gala’ apple at different development stages, and constructed an apple cDNA library along with Takara Biotechnology Co., Ltd. to be used in yeast systems. We incorporated the *MdMYB10* promoter sequence into the *pABAi* vector. The recombinant plasmid was linearized with *Bstb1* endonuclease and transformed into Y1H yeast cells, which were grown on -Ura selection medium in plates. The optimal 3-Amino-1, 2, 4-Triazole (3-AT) concentration for inhibiting autoactivation was determined. Additionally, yeast competent cells containing the bait vector were prepared. After adding the cDNA library, a series of heat shock and ice bath treatments was completed before positive clones were selected on -Leu solid medium containing the optimal 3-AT concentration in plates. MdNAC1-pGADT7 *pro*MdMYB10-pHIS2 and *pro*MdUFGT-pHIS2 recombinant plasmids were constructed and transformed into Y187 yeast cells. The optimal 3-AT concentration was determined to inhibit background leakage on -Trp and -His deficient media. The interaction was detected by growth pattern on a medium lacking -Trp, -His and -Leu but containing 3-AT. Primer sequences were listed in Table S3 (see online supplementary material).

### Electrophoretic mobility shift assay(EMSA)

The MdNAC1- pET32a recombinant plasmid was used to transform *Escherichia coli* BL21 cells for purification of the His-tagged fusion protein. A DNA sequence labeled at the 3′ end was synthesized as a Hot probe. Mutant probes with different nucleotide mutations in the *cis*-acting element were designed, whereas synthetic unlabeled DNA sequences were used as competitor probes. The primer of EMSA was designed (Table S3, see online supplementary material) and the experiment was carried out according to the manufacturer’s instructions (www.thermoscientific.com/pierce).

### Dual-luciferase reporter gene detection and *in vivo* imaging analysis following an ABA treatment

The recombinant NAC1- pGreenII 62-SK plasmid was incorporated into *A. tumefaciens* GV3103 cells along with P19 as an effector. The *MdMYB10* and *MdUFGT* promoter were constructed into the *pGreenII 0800-LUC* vector for the transformation of *A. tumefaciens* cells with the reporter constructs. *A. tumefaciens* was injected into tobacco leaves. The luminescence intensity was detected using a dual-luciferase reporter gene detection system (NightOWL II LB983; Berthold Technologies, Germany). To analyse the effects of ABA, the underside of the transiently transformed tobacco leaves were sprayed with 40 μM ABA. The imaging analysis was then performed as described above. Information regarding the primers used was listed in Table S3 (see online supplementary material).

### Amino acid sequence alignment and domain analysis

We screened the National Center for Biotechnology Information database (https://www.ncbi.nlm.nih.gov/) for sequences that were highly homologous to MdNAC1 in different species. The alignment function of the DNAMAN software was used to align the amino acid sequences of different species, whereas the MEGA5.1 software was used to construct a phylogenetic tree for the NAC family members in various species. The NAC1 conserved domains were analysed using an online program (https://meme-suite.org/meme/tools/meme). The relevant gene sequences are provided in the File S1 (see online supplementary material).

### Yeast two-hybrid assay

NAC1-pGADT7 and bZIP23-pGADT7 recombinant plasmids were constructed. NAC1 sequence was divided into two parts (NAC1^-N^-BK and NAC1^-C^-BK) and inserted into *pGBKT7* vector, respectively. The complete *NAC1* sequence was also inserted into the *pGBKT7* vector. The recombinant *pGBKT7* plasmids were inserted into Y2H Gold yeast cells, after which self-activation was assessed on medium lacking -Trp and -Leu The *pGADT7* and *pGBKT7* recombinant plasmids used for analysing protein–protein interactions were incorporated into yeast cells that were then grown on medium lacking -Trp, −Leu, -His, and -Ade. The recombinant plasmid containing NAC1^-N^ -BK was used as the bait for screening the cDNA library. The NAC1^-N^ -BK and cDNA library plasmid were transformed into Y2H-Gold cells. Positive strains were screened on medium lacking -His and -Ade. The genes encoding proteins that interacted with the bait were sequenced.

### Production of transgenic apple calli

The transgenic apple callus was obtained using the previous method [77]. Briefly, the *NAC1* sequence with the stop codon deleted was inserted into the *pRI101-AN* vector containing the sequence encoding the GFP-tag, whereas the *bZIP23* sequence was inserted into the *pCB302* vector containing the sequence encoding the HA-tag. To obtain NAC1-RNAi transgenic calli, 420-bp sense and antisense *MdNAC1* sequences containing different restriction sites were inserted into the *pFGC-1008* vector. All three vectors contained the 35S promoter sequence. The constructed recombinant plasmids were used to transform *A. tumefaciens* LBA4404 cells. At 20–30 min after the *A. tumefaciens*-mediated transformation, the calli were transferred to MS solid medium lacking antibiotics and cultured at 24°C for 1–2 days. Finally, the transformed calli were transferred to screening medium containing different antibiotics.

### Transient transformation of apple fruits

The *NAC1* coding sequence was inserted into the *PHB-GFP* vector to obtain the NAC1-PHB overexpression recombinant plasmid. The *NAC1* sequence was inserted into the TRV2 vector to obtain the NAC1-TRV2 suppressed expression viral recombinant plasmid, with *TRV1* used as the helper plasmid. The constructed recombinant plasmids and helper plasmid were incorporated into LBA4404 cells, which were then injected into apple fruit peels according to the required experimental combinations. The samples were kept in a light incubator at 22°C with a light intensity of 3000 lux for 3–5 days. The peel at the injection site was collected for an analysis of the anthocyanin content and real-time fluorescence quantification.

### Plant total RNA extraction and RT-qPCR analysis

Total RNA required for this experiment was extracted and reverse transcribed into cRNA by the experimental method mentioned above [50]. The RT-qPCR analysis was conducted using the iCycler iQ5 system (Bio-Rad). Gene expression was calculated according to 2^-ΔΔCT^ method. The RT-qPCR primers (Table S3, see online supplementary material) were provided by Qingke Biotechnology Co., Ltd.

### Determination of the anthocyanin content

Anthocyanins were extracted and determined by the previous method [19]. It was determined by spectrophotometry.

### Bimolecular fluorescence complementation analysis

The recombinant plasmids bZIP23-pSPYCE-YFP and NAC1^-N^-pSPYNE-YFP were constructed and transformed into GV3103 cells containing P19. The two GV3101 strains with recombinant plasmids were mixed (equal proportions) and co-injected into tobacco leaves, with the YFP-C empty vector used as the control. After a 24 h incubation in darkness, fluorescence was detected by BX53F confocal laser scanning microscope (Olympus, Tokyo, Japan) at 488 nm.

### Pull-down assay

The *NAC1* and *bZIP23* coding sequences were inserted into the *pET32a* and *pGEX4T-1* vectors, respectively. The constructed recombinant plasmids were incorporated into *E. coli* BL21 cells to produce the MdNAC1-His and MdbZIP23-GST fusion proteins. The differentially tagged fusion proteins were mixed (equal proportions) and then incubated at 4°C for 12 h. The HIS-labeled Protein Purification kit (Clontech) was used for the pull-down assay as previously described (Liu et al., 2019). The obtained protein was incubated with anti-GST antibodies and analyzed in a western blot.

## Acknowledgments

W.L., Y.W., X.C., and N.W. were responsible for the conception and design of the experiment. W.L., Z.M., T.G., L.Y., Z.L., Q.Z., and S.Z. participated in and completed the experiment. H.F. and Z.Z. contributed to manuscript writing. All authors have read and approved the final version of the manuscript.

This study was supported by the Natural Science Foundation of Shandong Province (ZR2020QC144, ZR2022MC017), the National Natural Science Foundation of China (32002002) and the Key Research and Develop ment Programs of Shandong Provence (2021LZGC024).

## Data availability

The authors confirm that all data from this study are available and can be found in this article and in supplementary information.

## Conflict of interest statement

The authors declare no competing interests.

## Supplementary data

Supplementary data is available at *Horticulture Research* online.

## Supplementary Material

Web_Material_uhad049
