# Supplementary material for: The ABA-induced NAC transcription factor MdNAC1 interacts with a bZIP-type transcription factor to promote anthocyanin synthesis in red-fleshed apples
Source: Hortic Res. 2023 Mar 15;10(5):uhad049. doi: 10.1093/hr/uhad049 (PMC10186271; doi:10.1093/hr/uhad049)
Supplement: Web_Material_uhad049 [file web_material_uhad049.zip › Supplementary Figure.pdf]

GGTTCATGTATAACAGTATCAGTTCGTGCATCAGTGGTTCAATTGCAGTGCTCAGAAATC
GTTCGAAGGTCTAAGGTGACATAAATTCCCCCCTATTTCTGTTCGAAATCTTCAATTCTT
TAGATTTAAGGTATTCAGTTTTAGGGATATAGGCTTGAAGAATCAATTAGGGATTTACAAA
ATGATTAAAGGGATTTTGGGTGTTTGCTGTTGCCATTTTTGAACACAACATCAGTTCCAC
TACTCTTTCATTTTCCCTCAATTTCTGAGCAACCAAACAAGTAGCATTATTGACAACATAC
TGAGCTCCTCGTGTCAACCATTCTTGAAAGAATCCTAATAAAGATTTATAGGCAAATTATG
CCCTAGAAAAAAATTTAATAAAAAGGACCCTGAACACGTAGGAACCGGCCCGTTTGTAAC
AGACTGAGATAGGTCCGGTTCTATTTCTTAAAAACCCAACACCCGCTACGTTCCATTTAT
AAACGGGTCGGTCTGGTCCCTCCAACTTTGAGCCCGGCTCGACTTGTGCCCACTCCTA
AACTAAACCATATAAAAACCAAGATTTCCCTTTCATCTTTCACACATATCACGTTACTTTC
CAACAACAATTCAACAATCACAACAAATAATCAACCATCAAGATCATATATCACGTCACTA
ATAAAGACAACCTTCATAAGGGTTGCCGTAGTTCTCTACTTGAAATCCAATTGTCTAGCA
TTGTAACCCTAAGTTACAGACACAAACATAAACTTGAGCAACTTCTATGCATAAGAATCTA
GGGTTTTGGACTAACTCAACAGAACCTAACAAGAAATAATATTCTGGACCGCTTAACGG
AATCCAACGAAGACAAGGTTTCGGACCACTCAACGGAACAAATAAGGAAGGGATATAAA
CCATTCAACGAAATCCATCTTTAGAATACGCATAGTCCCCCAATACGGATTAACCAAGTG
AGAACATACGCCATCTGATAGCGTGGTCCCGCAAGACAGTTAACCAAGTAGGACCACC
GATGGTATAATGTGACCAAGTAAGCAGTGACCCTAAATGTAGATTAAACCACGTGGAGT
TAAATTAACAAGGCTGAACCACCTATGAAAATAATGTAAGCCTGAAATCTTAGGAGAGAA
TTCTTGCTCTAGGGGACAAATGATTTTCGTATGCCTAAGTGTTTTTTTAGTGACAGTAAA
CTAAGATTTGAGTACAGAGACATTAACTGAGATTGACTCTTGTGAAAGCTTAGTGAGTT
GAAGCACGTAGGCCAATTATATTGAGCAATGTGTTAGGTGTAGCGTCTAAACTTCCGTA
GGAGTTTTGTACAGCAATATAGTGGGGGTGCCGCAAAATGCAGACAGTAGCAATAAATT
ACGGGCTAGGATTTTCTCCTCTTTTTTTTTCGTTCCATTCCATCCATTCCTCTCACATTCT
TTATTTTGTCTTTCTCTTTCTATAAAAAAATTAATATAAGATGTTAATGTAACTTGACCGTGA
CTATTCAAATAGGAGGGGAATGAAGAAGAGGGAAAAAAAGAGAGGAGAGAATCCTACT
CCGTAAATTACAAGCAAACACTTTTTTTTTTTTTTGGACAAGCAGAAGCAAACAAACACT
TGAAAAAGCAGCGAAAGCATGATAAAGGTATCTTATGGTGGTCAAAGATGTGTGTTGTA
ACTAGTTACACGATTCTGCATTCACATTCATAGAATGTGCTTTTGAATATTATATTACAGCT
AGAGAATTTTATGCCCTGGGATTGATTTCCCTTGTCAATGTTGTCGTGCAGAAATGTTAG
ACTGGTAGCTATTAACAAGTTAGACTGGTTAGACGGGTAGCTATTAACAAGTTAGACTGG
TAGCTATTAACAACTGGTAGCTATTAACAAGTTAGACTGGTAGCTATTAACAAGTTAGACT
GTGTGTGTGTGTGTATTTCACAAGTTAGACTGGTAGCTATTAACAACTGTTGGAATGTTT
TAAACTTGTCAGTGTTTGCTTCTGTGGA

ACACGT **NAC-specific binding motif**

CACGT **ABRE motif**

TTTCGG **LTR motif**

CACGTG **G-box motif**

TTGACC **W-box motif**

CAACTG **MBS motif**

Figure S1 Analysis of cis-acting elements in *MYB10* promoter sequence. [ACACGT] sequence is a cis acting element specially combined by NAC family.

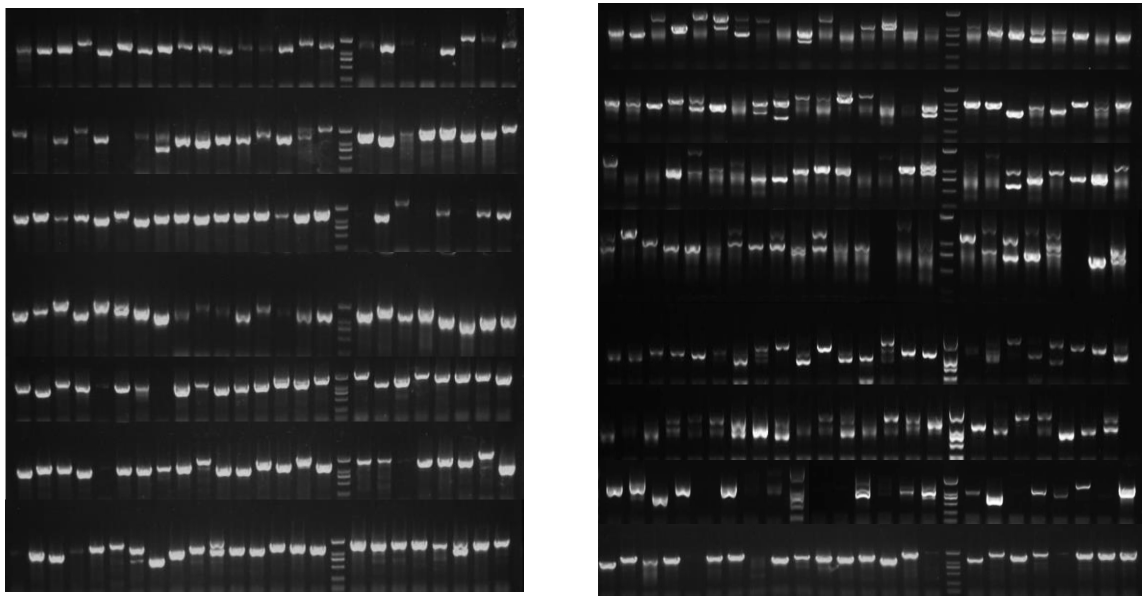

Figure S2 Screening candidate genes from yeast one rid cDNA libraries. The positive clones identified by qPCR.

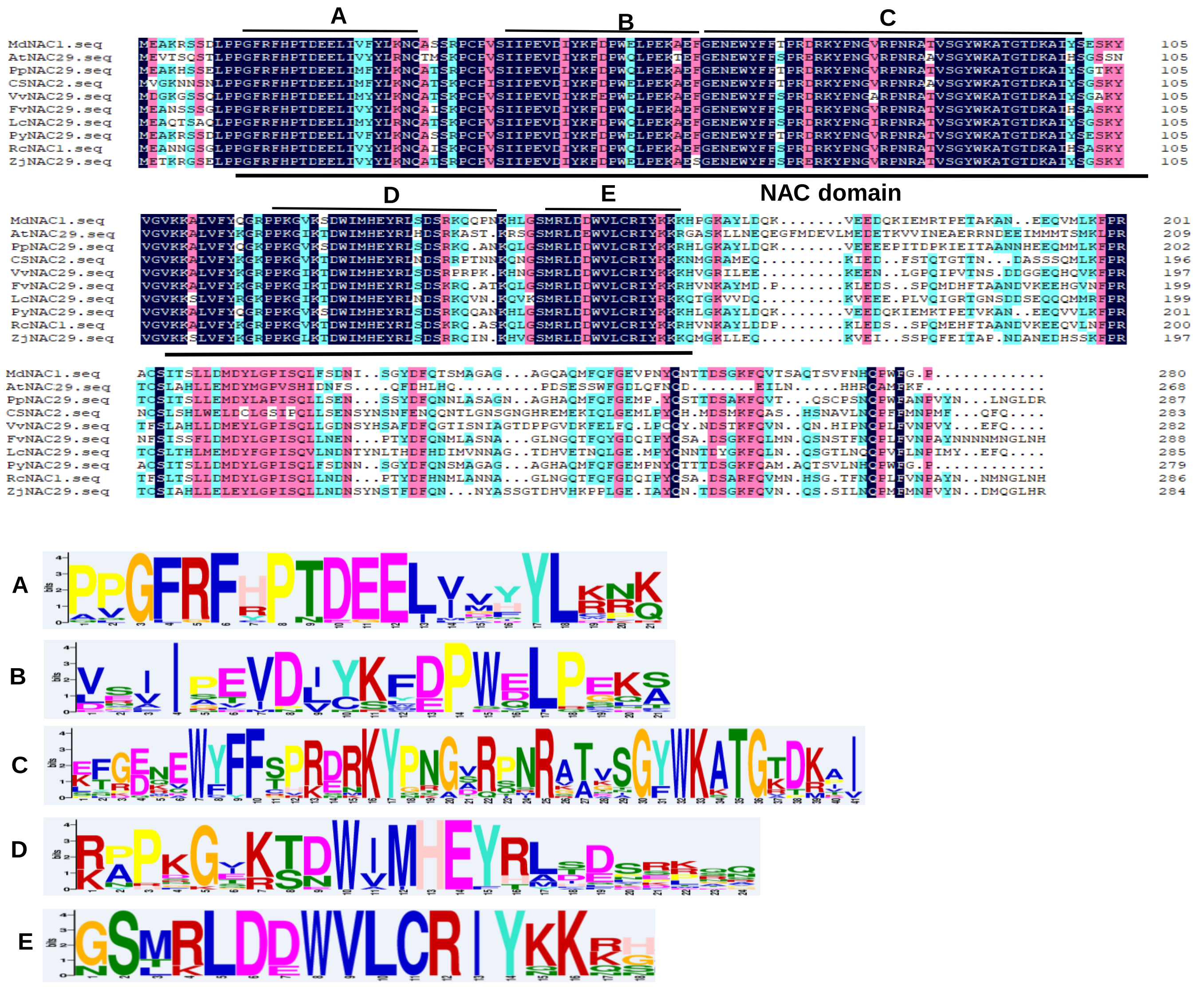

Figure S3 Biological analysis of MdNAC1 domain. The N-terminal of NAC1 is conservative in different species, and A B C D E is five relatively conservative domains. The protein serial number is obtained from NCBI,(https://www.ncbi.nlm.nih.gov/) *MdNAC1* : NM_001294010.1 ;*CsNAC2* XP_028086269.1 ; *FvNAC29*: XP_004297288.1 ; *LcNAC1*: UKF18673.1 ; PpNAC29 : XP_007223324.1 ; *PyNAC29* : KAB2624529.1 ;*AtNAC29* : XP_002887238.1 ; *RcNAC1* : XP_024192624.1 ; *VvNAC29* : ALP48443.1 ;*ZjNAC29* : XP_015895037.1 .

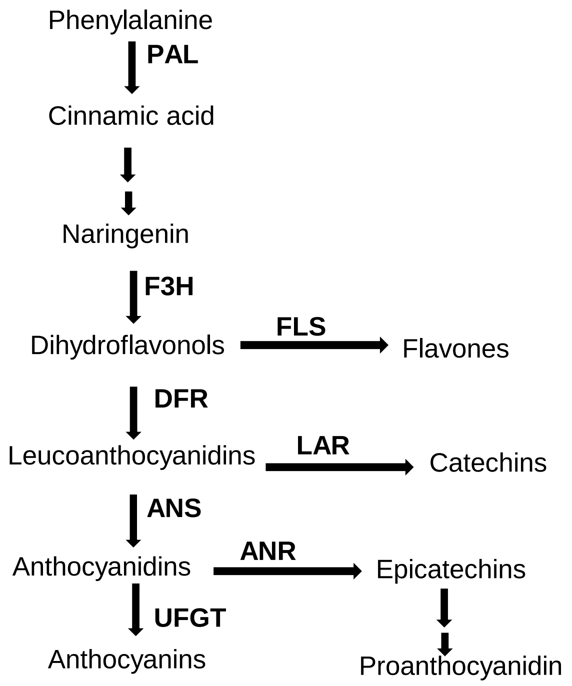

Figure S4 Schematic diagram of important structural genes in the anthocyanin synthesis pathway.
PAL: phenylalanine ammonia-lyase, *F3H*: flavanone 3-hydroxylase , *FLS*: flavonol synthase
DFR: dihydroflavonol 4-reductase, *LAR*: leucoanthocyanidin Reductase, *ANS*: anthocyanidin synthase,
ANR: anthocyanidin reductase, *UFGT*: UGP glucose-flavonoid 3-O-glucosyl

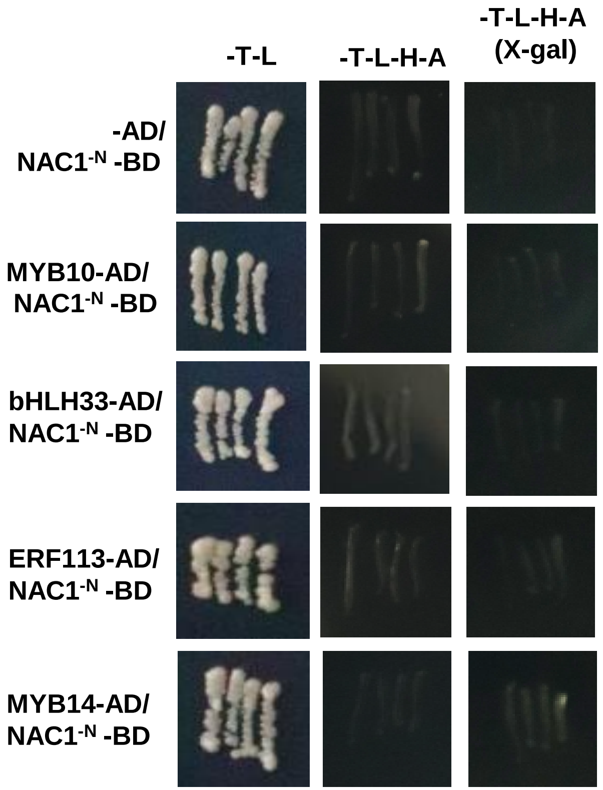

Figure S5 Y2H assays showing that MdNAC1 does not interact directly with MdMYB10, MdbHLH33, MdERF113 or MdMYB14.

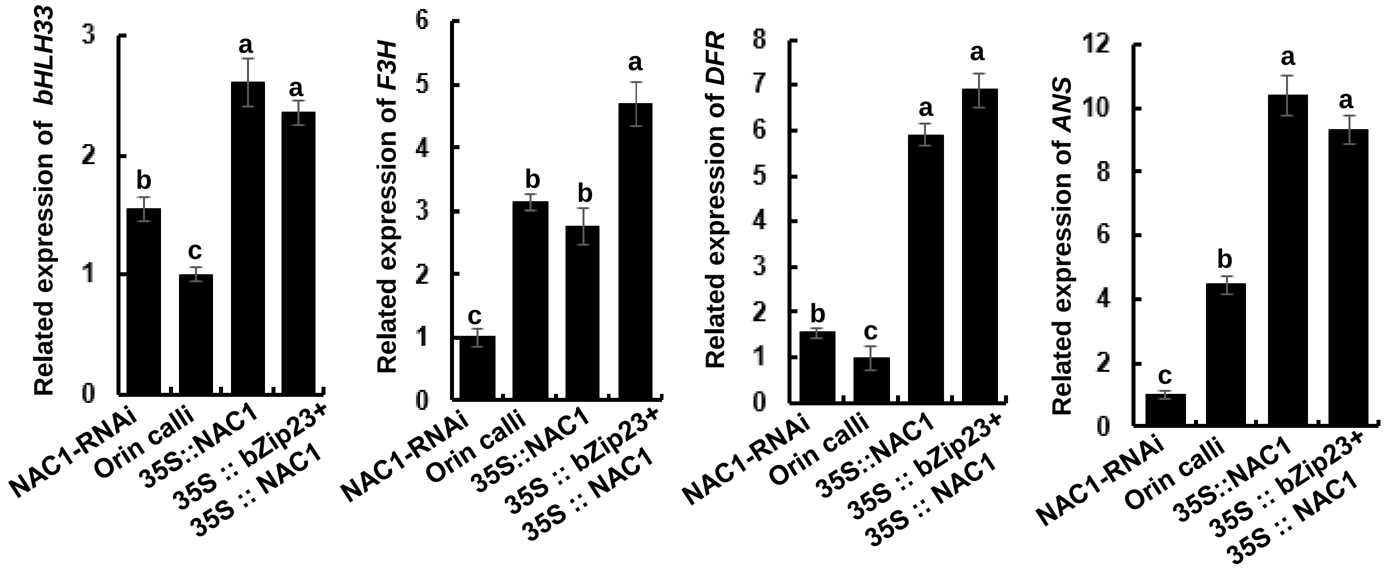

Figure S6 The expression level of *bHLH33 F3H DFR ANS* in different calli was determined by qRT-PCR. Values are means ±SD of three independent biological replicates, different letters indicate significant differences ($p < 0.05$).

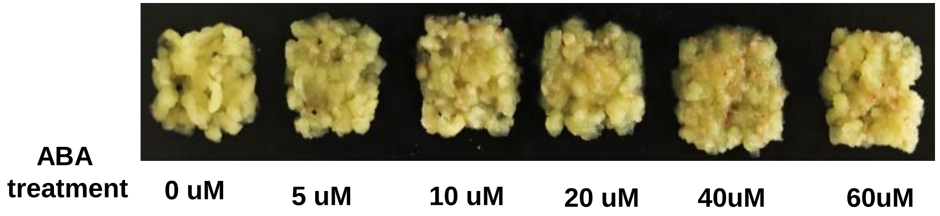

Figure S7 Treatment of orin callus with different concentrations of ABA. ABA of different concentrations was added to the MS based medium and exposed to light for 5-7d.

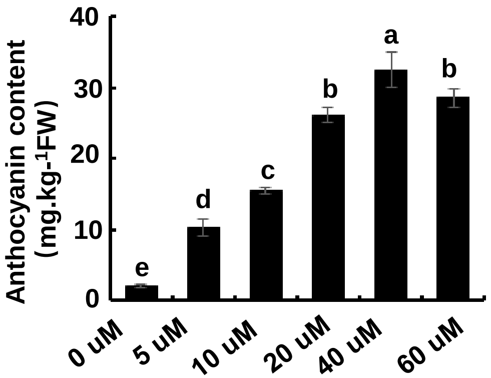

Figure S8 Anthocyanin content in Orin callus under different concentrations of ABA. Data are expressed as the means ± SD, n = 3. The different letters denote significant differences according to one-way analysis of variance (ANOVA) ($P < 0.05$)

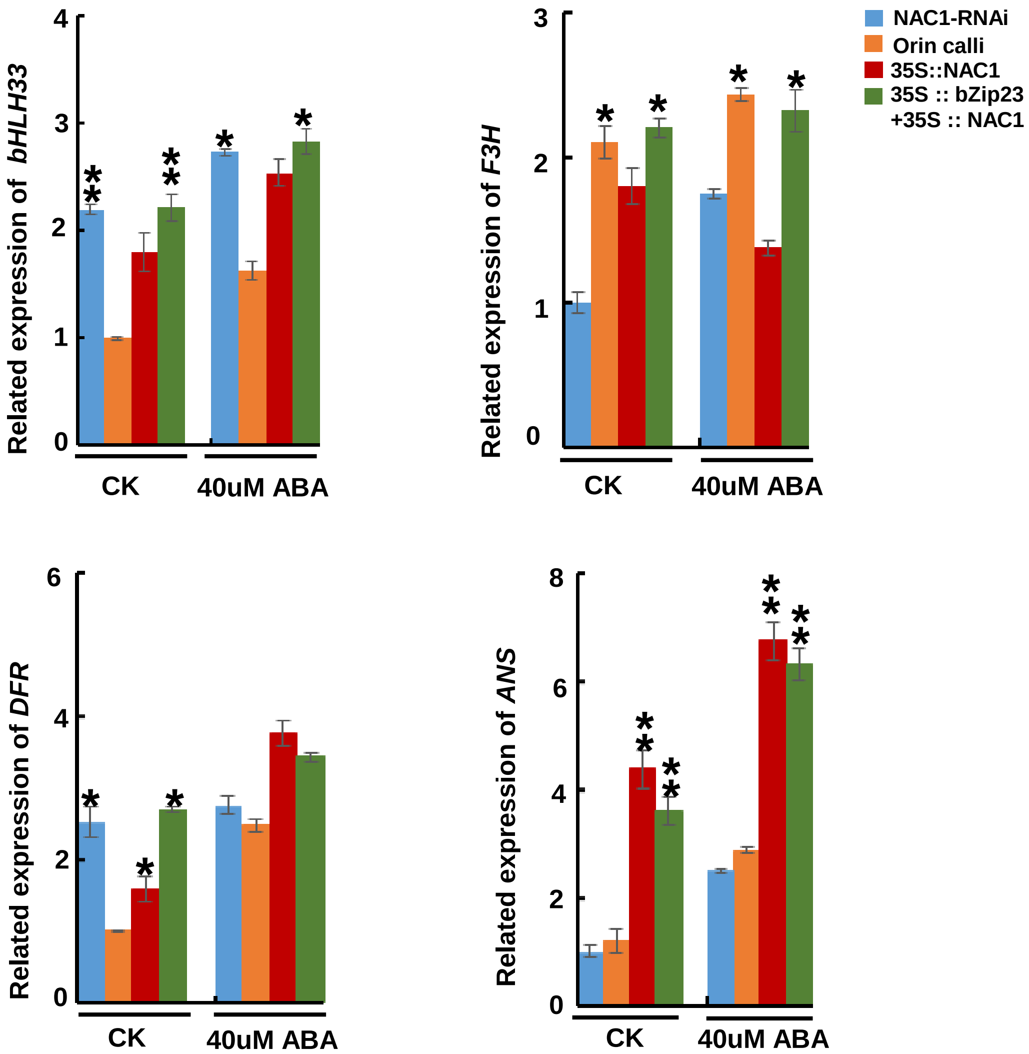

Figure S9 The expression level of *bHLH33 F3H DFR ANS* in different calli with or without ABA was detected by qRT-PCR. CK and 40uM ABA represent the presence or absence of ABA, respectively. Values are means ±SD of three independent biological replicates. Asterisks indicate statistical significance by Tukey's test using DPS software (*P < 0.05 and **P < 0.01).
